# Bidirectional substance P signaling between periodontal ligament fibroblasts and sensory neurons under mechanical stress

**DOI:** 10.3389/fnmol.2025.1583908

**Published:** 2025-05-16

**Authors:** Judit Symmank, Lara Löffler, Ulrike Schulze-Späte, Collin Jacobs

**Affiliations:** ^1^Department of Orthodontics, Jena University Hospital, Jena, Germany; ^2^Section of Geriodontics, Department of Conservative Dentistry and Periodontics, Jena University Hospital, Jena, Germany

**Keywords:** nociception, orthodontics, periodontal ligament fibroblasts, substance P, TAC1

## Abstract

**Introduction:**

Orthodontic tooth movement (OTM) and treatment-associated pain are closely related processes driven by a local inflammatory response modulated by periodontal ligament fibroblasts (PdLFs). Increased levels of substance P (SP), a well-characterized tachykinin, has been demonstrated in the PdL following the application of orthodontic forces. Although traditionally considered as neurotransmitter modulating inflammatory processes and pain, recent evidence suggests that also non-neuronal cells contribute to SP signaling during OTM. Since sensory neurons also express the corresponding receptor NK1R, activation by SP appears to be possible. However, the contribution of PdLFs to SP signaling upon mechanical stress and their subsequent interaction with sensory neurons remain largely unexplored. Thus, the aim of the study was to investigate a potential SP-mediated interactions between PdLFs and sensory neurons advancing our understanding of molecular mechanisms underlying orthodontic pain during OTM.

**Methods:**

TAC1 and SP levels were quantified via qRT-PCR, Western blot, and ELISA in compressed human PdLFs. Their conditioned medium was applied to sensory-like SH-SY5Y neurons and their activation was assessed by morphological features, cFOS expression, and calcium influx. Conversely, PdLFs were stimulated with conditioned medium from capsaicin-activated SH-SY5Y neurons. Subsequently, cytokine expression, RANKL/OPG ratio and activation of immune cells and osteoclasts by PdLFs were evaluated.

**Results:**

Compressive force induced a time- and intensity-dependent increase in TAC1 expression and SP secretion by compressed PdLFs with a peak at 24 h. Stressed PdLFs significantly increased neurite complexity, cFOS levels and calcium influx in sensory neurons, indicating their activation. Conversely, activated neurons elicited a robust pro-inflammatory response in PdLFs along with an increased osteoclastogenesis.

**Discussion:**

Our findings demonstrate that PdL fibroblasts could function as a novel non-neuronal source of SP modulating sensory neuron activation. Conversely, fibroblasts were also stimulated by SP effecting inflammation and osteoclastogenesis. These findings underscore a dynamic role of PdLF- and sensory neuron-derived SP that likely contributes to both pain perception and inflammatory bone remodeling during OTM.

## 1 Introduction

Orthodontic treatment is frequently associated with significant pain, despite its substantial benefits for oral health and functionality (Long et al., 2016). Clinically, orthodontic pain typically peaks within 24 h after treatment steps such as appliance activation or routinely performed adjustments (Erdinç and Dinçer, 2004; Marković et al., 2015). This negatively influences health quality and compliance of patients impacting treatment duration and success (Long et al., 2016). The underlying mechanism of pain perception involves the activation of nociceptive sensory neurons within the periodontal ligament (PdL), a connective tissue that anchors the tooth to the alveolar bone (Wang et al., 2024). This tissue is highly innervated by sensory nerve fibers originating primarily from the trigeminal nerve (Harris, 1975; Linden, 1990). Consisting of Aδ- and C-fibers, they play a critical role in transmitting nociceptive signals to the central nervous system (Toda et al., 2004).

Orthodontic pain and tooth movement are two interrelated and interdependent events whose common biological mechanism includes an aseptic local inflammation initiated by cells of the PdL, mainly fibroblasts (PdLFs) (Morii et al., 2020; Toyama et al., 2023). Compressed PdLFs promote a pro-inflammatory microenvironment via secretion of diverse cytokines including interleukin-1 beta (IL-1β), IL-6, tumor necrosis factor alpha (TNF-α), and cyclooxygenase-2 (COX-2) (Inchingolo et al., 2024; Stemmler et al., 2021). Besides stimulating immune and bone-remodeling cells (Behm et al., 2022; Nakai et al., 2023), these inflammatory signals may also contribute to nociceptive sensitization and link PdLF-mediated responses to orthodontic pain (Long et al., 2016; Toyama et al., 2023). In addition, compressed PdLFs secrete increasing levels of receptor activator of NF-κB ligand (RANKL), which binds to its receptor RANK expressed by osteoclast precursors (Symmank et al., 2020). Osteoprotegerin (OPG) acts as decoy receptor of RANKL and its secretion by PdLFs is reduced upon mechanical compression (Kanzaki et al., 2019). Consequently, the mechanical stress-induced increase in the RANKL/OPG ratio facilitates osteoclast differentiation and bone resorption, which are critical parameter in orthodontic tooth movement (OTM) (Yamaguchi, 2009).

Beyond their role in sensory transmission, innervating sensory neurons actively modulate local inflammatory responses via neuropeptide release (Norevall et al., 1995; Yamaguchi et al., 2004). Tachykinins are a family of neuropeptides, with substance P (SP, gene: *TAC1*) as one important mediator in pain processing and inflammatory regulation (Steinhoff et al., 2014). Traditionally, SP is recognized as neurotransmitter released from sensory neurons, which exerts its effects primarily through the neurokinin-1 receptor (NK1R) expressed in diverse cell types (Navratilova and Porreca, 2019). Activation of NK1R by SP leads to the release of pro-inflammatory cytokines, vasodilation, and increased vascular permeability, contributing to an inflammatory response observed during OTM (Sacerdote and Levrini, 2012). SP has been detected in gingival crevicular fluid of orthodontic patients (Levrini et al., 2013; Yamaguchi et al., 2009), as well as in the PdL and dental pulp of experimental orthodontic animal models (Caviedes-Bucheli et al., 2021; Nicolay et al., 1990; Norevall et al., 1995), with levels peaking 24 h after the application of orthodontic force. This temporal pattern correlates with pain perception reported by orthodontic patients (Erdinç and Dinçer, 2004; Marković et al., 2015).

Although traditionally considered a neurotransmitter, recent evidence suggests that non-neuronal cells may also contribute to increasing SP levels during OTM. Specifically, dental pulp fibroblasts (DPFs) have been demonstrated to upregulate SP expression in response to mechanical forces (Caviedes-Bucheli et al., 2021). Given these findings and the previously observed increase in SP in the PdL (Nicolay et al., 1990; Norevall et al., 1995), we hypothesize that PdLFs may serve as an additional, non-neuronal source. In view of the observed NK1R expression in sensory neurons (Butler et al., 2018), specifically also in trigeminal Aδ- and C-fibers (Edvinsson et al., 2021), we therefore propose that fibroblast-derived SP may contribute to their activation, possibly influencing pain perception. Thus, the aim of this study is to investigate potential SP-mediated interactions between PdLFs and sensory neurons, with a particular focus on inflammatory responses and osteoclastogenesis during OTM.

## 2 Materials and methods

### 2.1 Cell culture

Human periodontal ligament fibroblasts (hPdLFs, Lonza) were used as a pooled mix of four batches of healthy donors with different origin (50:50 Afroamerican:Eurasian), gender (50:50 men/woman), and age (26.75 years ± 5.88). They were cultured in Dulbecco’s modified Eagle medium (DMEM) with 4.5 g/L glucose (Capricorn Scientific), supplemented with 10% fetal bovine serum (FBS; Thermo Fisher Scientific), 1% penicillin/streptomycin (Thermo Fisher Scientific), and 1% L-ascorbic acid (Merck Millipore). Cells were maintained at 37°C with 5% CO_2_ and subculturing was performed at 75% confluency by trypsination. Passages four to eight were used for all experiments, as a fibroblastic phenotype with full differentiation potential is reliably maintained at these passages (Marchesan et al., 2011). SH-SY5Y neuroblastoma cells (Merck Millipore) were cultured in growth medium with consist of DMEM/F12 medium supplemented with 10% FBS, 2% non-essential amino acids (NEAA) and 1% penicillin/streptomycin (all Thermo Fisher Scientific) at 37°C with 5% CO_2_. Subculturing was performed prior 75% confluency by trypsination. During media changes and subcultivations, any non-adherent growing cells were pelleted and transferred to the new cultures. Cells from passages 13–15 were used for all experiments, as the ability to differentiate to neurons is reduced at later passages (Shipley et al., 2016). For neuronal differentiation, cells were treated with 10 μM retinoic acid (Merck Millipore) in differentiation medium A (DMEM/F12 with 2% FBS, 2% NEAA and 1% penicillin/streptomycin) for five days, followed by 50 ng/mL brain-derived neurotrophic factor (BDNF; Merck Millipore) in differentiation medium B (DMEM/F12 with 0% FBS, 2% NEAA and 1% penicillin/streptomycin) for two days. Monocytic THP1 cells (DSMZ) were cultured in RPMI 1640 medium (Capricorn Scientific) supplemented with 10% FBS and 1% penicillin/streptomycin at 37°C with 5% CO_2_. Subculturing was performed weekly.

### 2.2 siRNA transfection and mechanical stimulation of hPdLFs

Prior knockdown of TAC1, hPdLFs were seeded at 1 × 10^5^ cells per well in 6-well plates and cultured until 75% confluency. To induce a reduction in TAC1-derived substance P, hPdLFs were transfected with *TAC1*-targeting siRNA (Santa Cruz Biotechnology) via lipofectamine 2000 (Thermo Fisher Scientific) according to the manufacturer’s protocol. Subsequently, a static compressive force of 2, 4, or 6 g/cm^2^ was applied using sterile glass plates for 6 h, 24 h, or 48 h. Uncompressed cells served as controls. Conditioned medium was isolated and stored at −20°C. Cells were isolated for RNA and protein analysis.

### 2.3 Stimulation of SH-SY5Y neurons in transwell chambers

To assess the impact of mediators secreted by stressed hPdLFs, 2.5 × 10^4^ SH-SY5Y cells were seeded in the upper part of transwell chambers (growth area 33 mm^2^, pore size 3 μm; Millipore) and differentiated into sensory-like neurons as described before. Subsequently, neuronal processes extended through the pores into the lower chamber were exposed to conditioned medium of compressed hPdLFs for 30 min. To block NK1R signaling in SH-SY5Y cells, aprepitant was applied within the conditioned medium. Based on dose-response curves we established, which identified 28.5 μM as the IC_50_ regarding cell viability, 15 μM aprepitant was used for stimulation of differentiated SH-SY5Y neurons.

### 2.4 Quantitative polymerase chain reaction (qPCR)

Expression analysis was performed as recently described (Symmank et al., 2021). Briefly, RNA was extracted using TRIzol Reagent (Thermo Fisher Scientific) and the RNA Clean & Concentrator-5 kit (Zymo Research) according to the manufacturer’s protocols. Synthesis of cDNA was performed using SuperScript IV Reverse Transcriptase (Thermo Fisher Scientific) according to the manufacturer’s protocols. Gene expression was analyzed by qPCR with Luminaris Color HiGreen qPCR Master Mix (Thermo Fisher Scientific) using the qTower^3^ system (Analytik Jena) according to the manufacturer’s protocols. Primer were tested for specificity and efficiency as previously described (Symmank et al., 2021). Primer sequences are listed in Table 1. *RPL22* and *TBP* served as reference genes validated by Kirschneck et al. (2017). The ΔΔCT method was used for relative quantification.

**TABLE 1:** qPCR primer sequences of human genes indicated in 5‘–3‘ direction. bp, base pairs.

Gene	Gene symbol	NCBI gene ID	Primer sequence
Interleukin 1 beta	*IL1B*	3553	fw: CGAATCTCCGACCACCACTA rev: AGCCTCGTTATCCCATGTGT
Interleukin 6	*IL6*	3569	fw: CATCCTCGACGGCATCTCAG rev: TCACCAGGCAAGTCTCCTCA
Prostaglandin-endoperoxide synthase 2	*PTGS2* (alias *COX2*)	4743	fw: GATGATTGCCCGACTCCCTT rev: GGCCCTCGCTTATGATCTGT
Ribosomal protein L22	*RPL22*	6146	fw TGATTGCACCCACCCTGTAG rev GGTTCCCAGCTTTTCCGTTC
TATA box binding protein	*TBP*	6908	fw CGGCTGTTTAACTTCGCTTCC rev TGGGTTATCTTCACACGCCAAG
Tumor necrosis factor	*TNFA*	7124	fw CACGCTCTTCTGCCTGCTG rev AGGCTTGTCACTCGGGGTT
TNF receptor superfamily member 11b	*TNFRSF11B* (alias *OPG*)	4982	fw: GAAGGGCGCTACCTTGA rev: GCAAACTGTATTTCGCTC
TNF superfamily member 11	*TNFSF11* (alias *RANKL*)	8600	fw: ATCACAGCACATCAGAGCAGA rev: TCACTTTATGGGAACCAGATGGG

### 2.5 Enzyme-linked immunosorbent assay (ELISA)

To quantify the secretion of TAC1-associated tachykinins (mainly substance P and neurokinin A), cell culture supernatants from hPdLFs were analyzed via ELISA (Aviva Systems Biology, OKEH00417) following the manufacturer’s protocols. For specific analysis of substance P secretion by hPdLFs and SH-SY5Y neurons, a SP-specific ELISA with only low cross-reactivity with neurokinin A was conducted according to the manufacturer’s protocols (Abcam, ab288318).

### 2.6 Western blot

To analyze the protein levels of TAC1, hPdLFs were isolated by washing with ice-cold phosphate-buffered saline (PBS) and centrifugation. Protein isolation and expression analysis by semi-dry Western blot was performed as previously described (Symmank et al., 2018). The polyclonal rabbit-anti-TAC1 antibody (1:500, Thermo Fisher Scientific, Carlsbad) and goat-anti-rabbit IgG horseradish peroxidase (HRP; 1:2000, Thermo Fisher Scientific) were used to detect TAC1.

### 2.7 Immunofluorescent staining

To visualize protein expression and neuron morphology, immunofluorescent stainings was performed as recently described (Lösch et al., 2023). Briefly, cells were fixed in 4% paraformaldehyde (PFA), permeabilized with 0.1% Triton X-100 in PBS, and unspecific binding sites were blocked with 4% bovine serum albumin (BSA). Primary antibodies were applied in PBS/0.1 Triton X-100 and 4% BSA for 3 h at room temperature (RT) followed by washing steps in PBS/0.1 Triton X-100. Subsequently, Alexa Fluor-conjugated secondary antibodies (Thermo Fisher Scientific) were applied for 30 min at RT. Following primary antibodies were used: mouse-anti-NeuN (1:500, Cell Signaling Technology), rabbit-anti-CGRP (1:250, Thermo Fisher Scientific), mouse-anti-NK1R (1:250, Thermo Fisher Scientific) and rabbit-anti-cFOS (1:500, Thermo Fisher Scientific). Following secondary antibodies were used: Cy3-anti-mouse IgG, Alexa488-anti-mouse IgG and Cy3-anti-rabbit IgG (1:1000, all Jackson Immuno Research). Alexa647- or Alexa488-labeled phalloidin was used to stain β-actin (Thermo Fisher Scientific). Nuclei were counterstained with DAPI (1:10,000 in PBS; Thermo Fisher Scientific). Samples were embedded in Mowiol 4-88 (Carl Roth) and stored at 4°C until imaging.

### 2.8 Calcium imaging with Fluo-4 AM

To assess neuronal activation by hPdLFs, SH-SY5Y cells were loaded with Fluo-4 AM according to the manufacturer’s protocols (Thermo Fisher Scientific, F10489). Briefly, cells were shortly washed with serum free culture medium and subsequently incubated with Fluo-4 AM for 30 min at 37°C and 15 min at room temperature. After removing Fluo-4 AM loading solution, cells were washed ones in pre-warmed PBS. Conditioned medium of hPdLFs was simultaneously added to all cells and calcium flux was directly recorded for 2 min at 30 s intervals using a Zeiss Primovert microscope (Carl Zeiss Company). We used the neuro backdrop background suppressor solution included in the Fluo-4 AM loading kit.

### 2.9 THP1 activation assay

Upon stimulation, non-adherent monocytic THP1 cells can differentiate into adherent macrophages. To analyze the activation of monocytic THP1 cells by hPdLFs, 2.5 × 10^4^ THP1 cells labeled with CellTracker™ CMFDA (Thermo Fisher Scientific) were stimulated with the conditioned medium of hPdLFs. After 30 min, non-adherent cells were removed by washing with pre-warmed PBS. Remaining adherent CMFDA-positive THP1 macrophages were fixed with pre-warmed 4% PFA and stored at 4°C until imaging.

### 2.10 Osteoclast differentiation assay

For analyzing the impact of hPdLFs on the activation of osteoclast differentiation, pre-stimulated THP1 cells were cultured with the conditioned medium of hPdLFs as previously performed (Lösch et al., 2023; Nitzsche et al., 2024). Briefly, THP1 cells were differentiated into macrophage-like cells using 100 ng/mL phorbol 12-myristate 13-acetate (PMA, Merck Millipore) for two days and stimulated with conditioned medium of hPdLFs for six days. The conditioned medium was applied 1:1 with fresh THP1 culture medium. Cells were fixed with prewarmed 4% PFA, followed by tartrate-resistant acid phosphatase (TRAP) staining as performed previously (Lösch et al., 2023). Briefly, after post-fixation staining solution consisting of 0.1 mg/mL Naphthol AS-MX phosphate, 0.5 mg/mL Fast Red Violet LB salt, 1% N,N-dimethyl formamide in 50 mM sodium acetate trihydrate, 50 mM tartrate dehydrate and 0.1% acetic acid (all Merck Millipore) was applied for 60 min at 37°C to label TRAP-positive cells in purple. Subsequently, counterstaining of nucleic acids with SYTO (Thermo Fisher Scientific) was performed for 5 min. Cells were washed with PBS and directly imaged.

### 2.11 Microscopy

Immunofluorescent stainings were imaged using a BZ-X800 (Keyence). Fluo-4 AM levels, TRAP/SYTO staining and THP1 activation were imaged using a Zeiss Primovert microscope (Carl Zeiss Company).

### 2.12 Morphological analysis

Cellular complexity of SH-SY5Y neurons was analyzed with Fiji software^1^ (version number 1.52p) as previously described (Symmank et al., 2018). It is defined by the product of the number of neurites, the branch points of the longest neurite and the length of the longest process. It is displayed as relative cellular complexity in relation to SH-SY5Y stimulated with the conditioned medium of control hPdLFs.

### 2.13 Immunofluorescence intensity analysis

Immunofluorescence intensities of cFOS and Fluo-4 AM were analyzed with Fiji software^1^ (version number 1.52p) as previously reported (Symmank et al., 2018). Microscopic imaging was conducted under standardized settings across all experimental conditions, ensuring consistency. Imaging of all conditions of one experiment was performed on the same day using pre-warmed lasers and identical scanning parameters. To prevent overexposure, imaging parameters were adjusted based on the most intense condition. Mean gray values (MGVs) for cFOS and Fluo-4 AM were quantified in the cytoplasm of 180 cells per condition, with background correction applied as previously described (Schuldt et al., 2022a; Schuldt et al., 2022b). To account for the variability between experiments, the MGVs of the treated conditions were normalized to the mean of the MGVs of the respective control condition within each experiment, resulting in relative fold changes. The mean values of all experiments are displayed in the respective diagrams. MGV intensities were represented using a thermal LUT.

### 2.14 Histochemical analysis

Osteoclast activation was assessed by identifying multinucleated TRAP-positive cells by overlaying TRAP and SYTO staining. The data is displayed in relation to the control as fold change.

### 2.15 Statistics

Statistical analyses were conducted using GraphPad Prism 10.4.2 (GraphPad Software). Normality of data distribution was evaluated using the Shapiro–Wilk test or the Kolmogorov–Smirnov test. Data are presented as mean ± standard error of the mean (SEM) from at least three independent experiments performed in technical duplicates. One-way and Two-way ANOVA with Tukey’s *post-hoc* test was used to compare multiple conditions. Significance levels: */#/§*p* < 0.05, **/##/§§*p* < 0.01, ***/###/§§§*p* < 0.001.

## 3 Results

### 3.1 TAC1 expression/substance P secretion is increased in compressed hPdLFs

**FIGURE 1:**
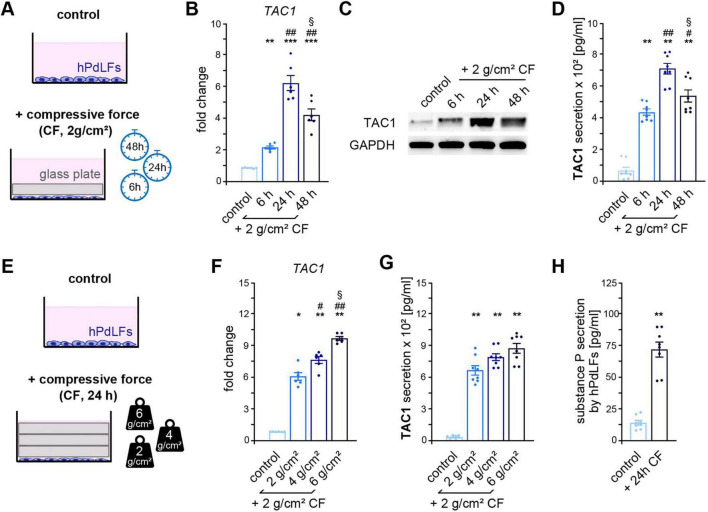
Mechanically compressed PdL fibroblasts show a duration- and intensity-dependent expression and secretion of TAC1 and substance P. **(A)** Experimental model illustrating the stimulation of human periodontal ligament fibroblasts (hPdLFs) with a compressive force (CF) of 2 g/cm^2^ for 6, 24, and 48 h using a sterile glass plate. **(B)** Quantitative expression analysis of *TAC1* in hPdLFs stimulated with increasing durations of CF. The expression levels are displayed in relation to the unstimulated control. **(C)** Protein expression levels of TAC1 in hPdLFs stimulated with increasing durations of CF. **(D)** Secretion levels of TAC1-derived neuropeptides, specifically substance P and neurokinin A, detected in the medium of hPdLFs stimulated with increasing durations of CF. **(E)** Experimental model illustrating the stimulation of hPdLFs with a compressive force of 2 g/cm^2^, 4 g/cm^2^ and 6 g/cm^2^ for 24 h using sterile glass plates. **(F)** Quantitative expression analysis of *TAC1* in hPdLFs stimulated with increasing CF. The expression levels are displayed in relation to the unstimulated control. **(G)** Secretion levels of TAC1-derived neuropeptides detected in the medium of hPdLFs stimulated with increasing CF. **(H)** Secretion levels of SP in the medium of hPdLFs stimulated with a CF of 2 g/cm^2^ for 24 h. */#/§ *p* < 0.05; **/## *p* < 0.01; *** *p* < 0.001; */**/*** in relation to control, #/## in relation to 6 h of 2 g/cm^2^ CF in **(B,D)** and 24 h of 2 g/cm^2^ CF **(F,G)**, §§ in relation to 24 h of 2 g/cm^2^ CF in **(B,D)** and 24 h of 4 g/cm^2^ CF **(F,G)**. One-way ANOVA with *post-hoc* test (Tukey’s). Results are shown as mean ± SEM with individual values.

To investigate the impact of compressive force on TAC1 levels in human periodontal ligament fibroblasts (hPdLFs), a compressive force of 2 g/cm^2^ was applied to an *in vitro* cell culture model for 6, 24, and 48 h (Figure 1A). Quantitative real-time PCR (qRT-PCR) revealed a significant time-dependent increase in *TAC1* expression peaking at 24 h (Figure 1B). In line, protein expression analysis demonstrated a time-dependent increase with a peak at 24 h of compressive stress (Figure 1C). Secretion of TAC1-associated proteins SP and NKA by compressed hPdLFs followed the TAC1 expression pattern, with the highest levels detected after 24 h of force application (Figure 1D). To assess whether TAC1 levels are influenced by the intensity of the applied compressive force, hPdLFs were subjected to forces of 2, 4, and 6 g/cm^2^ for 24 h (Figure 1E). *TAC1* expression levels increased dose-dependently, with the highest expression observed at 6 g/cm^2^ (Figure 1F). Similarly, secretion of SP and NKA by compressed hPdLFs showed a corresponding intensity-dependent increase, supporting the transcriptional findings (Figure 1G).

Given that the application of 2 g/cm^2^ for 24 h closely simulates physiological forces and aligns with the observed pain peak in patients (Erdinç and Dinçer, 2004; Marković et al., 2015), this condition was selected for subsequent experiments. To confirm the specificity of the force-induced increase in protein secretion, a SP-specific ELISA was performed verifying a significant force-dependent increase in SP secretion by compressed hPdLFs under these conditions (Figure 1H).

Together, these data indicate that compressive force leads to a time- and intensity-dependent increase in TAC1 expression and the secretion of SP by hPdLFs.

### 3.2 Fibroblast-derived substance P activates sensory-like neuronal cells

**FIGURE 2:**
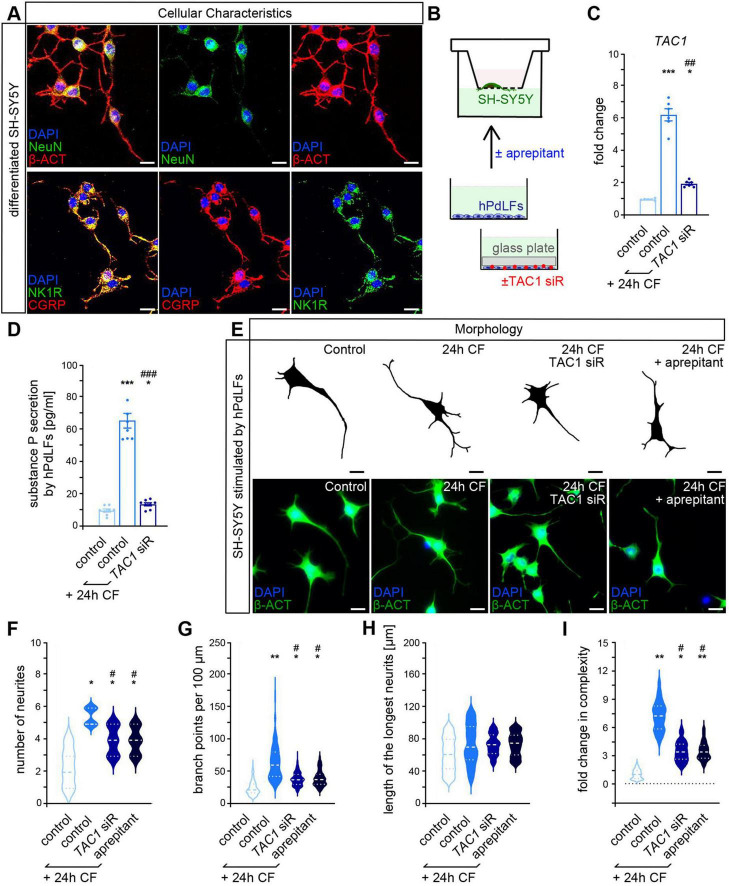
Sensory-like SH-SY5Y neurons are stimulated by compressed PdL fibroblasts in a substance P-dependent manner showing increased neuronal complexity. **(A)** Validation of the differentiation of SH-SY5Y cells to sensory-like neurons by immunofluorescent stainings of NeuN (green, upper panel) and CGRP (red, lower panel) with NK1R staining (green, lower panel), phalloidin-labeling of the actin cytoskeleton (β-ACT, red, upper panel) and DAPI for staining cell nuclei (blue). **(B)** Experimental model illustrating the stimulation of sensory-like SH-SY5Y neurons with conditioned medium of compressed hPdLFs. Downregulation of *TAC1* expression in hPdLFs was achieved by sequence-specific siRNA (*TAC1* siR). Blocking of NK1R in SH-SY5Y was performed by adding aprepitant to the conditioned medium. **(C)** Quantitative expression analysis of *TAC1* level in compressed hPdLFs treated with *TAC1* siRNA. **(D)** Secretion levels of SP by compressed hPdLFs treated with *TAC1* siRNA. **(E–I)** Visualization of the morphology of stimulated SH-SY5Y neurons by phalloidin-labeled (β-ACT, green, lower panel and traced in black, upper panel in E) and DAPI (blue, lower panel) for staining cell nuclei. Analyzed morphological parameters include the number of neurites from cell soma **(F)**, neurite branch points per 100 μm **(G)** and the length of the longest neurite **(H)** combined in the cellular complexity, which is shown in relation to the control in **(D)**. */# *p* < 0.05; **/## *p* < 0.01, ***/### *p* < 0.001; */**/*** in relation to control, #/##/### in relation to control +CF. One-way ANOVA with *post-hoc* test (Tukey’s). Scale bar: 50 μm in **(A)** and 25 μm **(C)**. Results are shown as mean ± SEM with individual values or distribution.

To evaluate the impact of fibroblast-derived SP for sensory neuron stimulation, human neuroblastoma SH-SY5Y cells differentiated into sensory-like neurons were utilized. Confirmation of successful differentiation was obtained by immunostaining for NeuN and CGRP with complementary staining of the actin cytoskeleton indicating neuron-like morphology (Figure 2A). Furthermore, expression of SP receptor NK1R was detected in differentiated SH-SY5Y cells.

To specifically investigate the effects of fibroblast-derived SP on sensory neuron processes, a transwell culture system was employed (Figure 2B). Differentiated neurons were cultured in the upper chamber with their neurites spreading through the porous membrane into the lower chamber. Neurites were then exposed to conditioned medium from hPdLFs subjected to 24 h of compressive force. This mimics the *in vivo* setting, in which only the innervating axons of the trigeminal neurons in the periodontal ligament are stimulated, but not their cell bodies. To determine the role of SP, *TAC1* expression was reduced by siRNA-mediated knockdown in hPdLFs (Figure 2C), which was accompanied by a reduced SP secretion (Figure 2D). Controls were treated with non-targeting siRNA. In addition, NK1R activity in differentiated SH-SY5Y neurons was inhibited by aprepitant, an SP receptor antagonist.

**FIGURE 3:**
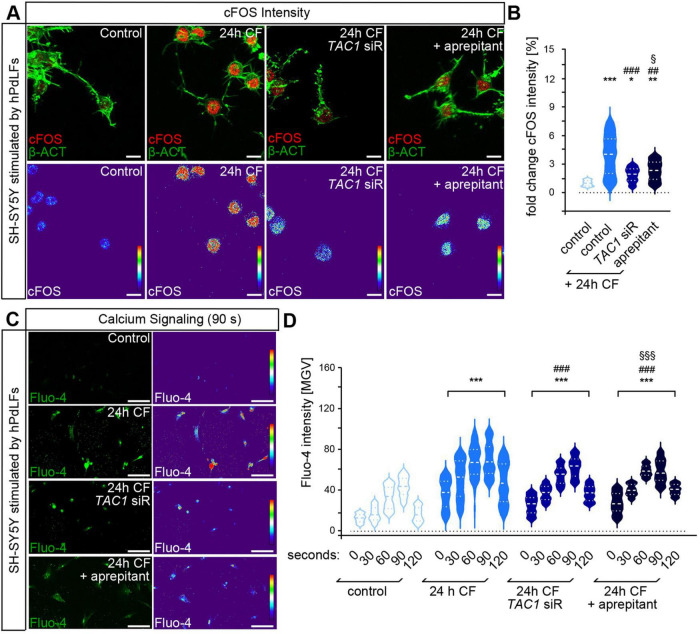
Compressed PdL fibroblasts activate sensory-like SH-SY5Y neurons in a substance P-dependent manner showing increased cFOS expression and calcium influx. **(A,B)** Immunofluorescent labeling of cFOS [red, upper panel in **(A)**] and phalloidin-labeling of the actin cytoskeleton [β-ACT, green, upper panel in **(A)**] in sensory-like SH-SY5Y neurons stimulated by conditioned medium of compressed hPdLFs. Downregulation of *TAC1* expression in hPdLFs was achieved by sequence-specific siRNA (*TAC1* siR). Blocking of NK1R in SH-SY5Y was performed by adding aprepitant to the conditioned medium. The intensity of cFOS is shown as thermal LUT [lower panel in **(A)**] and analyzed in relation to the control in **(B)**. **(C,D)** Fluorescent labeling of the calcium influx [Fluo-4, green, upper panel in **(C)**] in SH-SY5Y neurons 90 s after start of stimulation with conditioned medium of hPdLFs with the fluorescence intensity shown as thermal LUT [lower panel in **(C)**]. In **(D)** time points after stimulation start (0, 30, 60, 90, and 120 s) were analyzed in relation to the respective control condition for each time point. */# *p* < 0.05; **/## *p* < 0.01; ***/### *p* < 0.001; */**/*** in relation to control, ##/### in relation to control +CF. §/§§§ in relation to *TAC1* siRNA +CF. One-way and two-way ANOVA with *post-hoc* test (Tukey’s). Scale bar: 25 μm in **(A)** and 100 μm **(C)**. Results are shown as mean ± SEM with value distribution.

Morphological analysis was conducted as it can be interpreted as a parameter of neuronal activity (von Bohlen und Halbach, 2013). Sensory-like neurons stimulated with conditioned medium from compressed hPdLFs showed enhanced neurite numbers (Figures 2E, F) and branch points (Figure 2G), while neurite length was unaffected (Figure 2H). Consequently, this resulted in an increased neuronal complexity (Figure 2I), a product of the morphological parameters. This increase in complexity, as well as of the single parameters, neurite number and branch points, were diminished when *TAC1* expression was down-regulated in hPdLFs or when NK1R was inhibited in SH-SY5Y cells. To further assess neuronal activation, cFOS intensity was quantified using immunofluorescence, demonstrating a partial SP-dependent increase in neurons stimulated with conditioned medium from compressed hPdLFs (Figures 3A, B). This activation was reduced when *TAC1* was silenced in hPdLFs or NK1R was inhibited in SH-SY5Y cells. To confirm these findings, calcium signaling was monitored in stimulated SH-SY5Y immediately after administration of conditioned medium followed by a 30 s interval (Figures 3C, D). Under all conditions, the highest value was recorded 90 s after treatment start and decreased thereafter. Comparing all conditions, an increased calcium flux in sensory-like neurons treated with conditioned medium of compressed hPdLFs was detected, which was diminished by *TAC1* knockdown or NK1R inhibition.

In summary, our results emphasize that fibroblast-derived SP can stimulate sensory-like neurons via NK1R, suggesting a role for SP in mediating fibroblast-neuron interactions in stressful situations.

### 3.3 Neuron-derived SP induces a pro-inflammatory response of PdL fibroblasts with enhanced osteoclast activation

**FIGURE 4:**
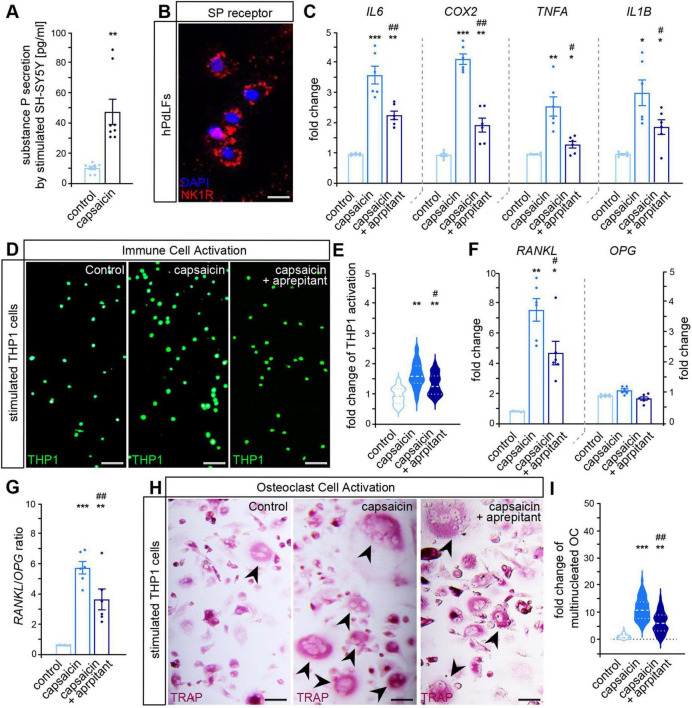
Activated sensory-like SH-SY5Y neurons SP-dependently induce a pro-inflammatory response of PdL fibroblasts with stimulated osteoclastogenesis. **(A)** Secretion levels of substance P detected in the medium of sensory-like SH-SY5Y neurons stimulated with capsaicin. **(B)** Immunofluorescent staining showing the expression of NK1R (red) in hPdLFs with DAPI (blue) for staining of the nuclei. **(C)** Quantitative expression analysis of genes encoding pro-inflammatory cytokines in hPdLFs stimulated with conditioned medium of capsaicin-activated SH-SY5Y neurons. Aprepitant was used to block NK1R activity in hPdLFs. **(D,E)** Activation of CMFDA-labeled THP1 monocytic cells [green in **(D)**] by hPdLFs that were stimulated with the conditioned medium of SH-SY5Y neurons with or without aprepitant displayed in relation to the unstimulated control in **(E)**. **(F,G)** Quantitative expression levels of *RANKL* and *OPG* in hPdLFs stimulated with conditioned medium of capsaicin-activated SH-SY5Y neurons with or without aprepitant **(F)** displayed as value in **(G)**. **(H,I)** TRAP-staining of differentiated THP1 cells (purple) cultured with the medium supernatant of hPdLFs that were stimulated with the conditioned medium of SH-SY5Y neurons with or without aprepitant **(H)**. The mean number of multinucleated TRAP-positive cells per image is shown in **(I)**. */# *p* < 0.05; **/## *p* < 0.01; *** *p* < 0.001; */**/*** in relation to control, #/## in relation to stimulation with capsaicin-activated SH-SY5Y neurons. One-way ANOVA with *post-hoc* test (Tukey’s). Scale bar: 25 μm in **(B)** and 50 μm **(D,H)**. Results are shown as mean ± SEM with individual values or distribution.

Classified as neurotransmitter, SP is secreted by nociceptive sensory neurons exerting modulatory effects on diverse cells (Steinhoff et al., 2014). When stimulated with capsaicin, an established activator of nociceptive neurons (Frias and Merighi, 2016), sensory-like SH-SY5Y neurons demonstrated enhanced SP secretion (Figure 4A). In addition, immunofluorescent staining verified NK1R expression in hPdLFs (Figure 4B). To investigate the impact of neuron-derived SP, conditioned medium of capsaicin-activated SH-SY5Y cells was subsequently used to stimulate hPdLFs. Aprepitant was used to block NK1R in hPdLFs.

As a relevant part of the PdLFs stress response, we analyzed the expression of the pro-inflammatory cytokines *IL6*, *COX2*, *TNFA*, and *IL1B* (Figure 4C). Stimulation with SP-enriched conditioned media of SH-SY5Y neurons resulted in a significant increase in cytokine expression by hPdLFs, which was slightly reduced by aprepitant. However, cytokine levels remained higher than in the controls, implying that additional stimuli may contributed to the pro-inflammatory response. To functionally evaluate immune cell activation by hPdLFs, we assessed the differentiation of monocytic CMFDA-labeled THP1 cells cultured with the medium supernatant of stimulated hPdLFs (Figures 4D, E). Upon detecting pro-inflammatory signals, non-adherent monocytic THP1 cells differentiate into adherent macrophages and are therefore routinely used to display inflammatory processes (Schuldt et al., 2022a; Schuldt et al., 2022b; Stemmler et al., 2021). PdL fibroblasts stimulated with SP-enriched conditioned medium of sensory-like SH-SY5Y activated an enhanced number of THP1 cells to differentiate into adherent macrophages validating their increased pro-inflammatory response. This effect was partially attenuated by aprepitant, underlining the involvement of the SP/NK1R signaling pathway. However, even with aprepitant, THP1 activation levels remained elevated compared to controls.

We further investigated the role of SP in mediating PdL fibroblast-driven modulation of osteoclast differentiation, a critical process in bone remodeling. As a relevant activator of osteoclastogenesis, *RANKL* expression was significantly up-regulated in hPdLFs stimulated with SP-enriched conditioned medium from capsaicin-activated neurons, whereas *OPG* showed no significant changes (Figure 4F). Consequently, *RANKL*/*OPG* levels were therefore increased in SP-stimulated hPdLFs, suggesting an osteoclastogenic shift in their signaling (Figure 4G). The addition of aprepitant partially reduced *RANKL* expression and *RANKL*/*OPG* ratios but not to control levels, indicating the presence of SP-independent mechanisms. For functional validation, we assessed osteoclast differentiation by culturing pre-stimulated THP1 macrophages with supernatants from hPdLFs exposed to SP-enriched conditioned medium from SH-SY5Y neurons (Figures 4H, I). The formation of multinucleated, TRAP-positive osteoclasts was significantly increased under these conditions, further supporting the previous observation of a pro-osteoclastogenic response of SP-stimulated hPdLFs. Although treatment with aprepitant partially reduced osteoclast formation, the differentiation remained elevated compared to the controls.

These results demonstrate that SP acts as a critical mediator linking sensory neuron activation to inflammatory response by hPdLFs promoting a pro-osteoclastogenic microenvironment.

## 4 Discussion

Orofacial pain is a commonly reported adverse side effect of orthodontic treatment that may impact patient compliance and motivation to continue therapy (Long et al., 2016). However, pain perception varies considerably and appears to depend on patient and treatment-related factors (Alturki et al., 2024; Lorek et al., 2024). Therefore, deciphering cellular and molecular mechanisms involved in nociceptive processes is crucial for advancing our understanding of orthodontic pain. In this study, we identified a functional connection of periodontal ligament cells and sensory neuron afferents via the tachykinin substance P. Traditionally recognized as neurotransmitter, we identified PdL cells as additional non-neuronal source of SP upon mechanical stimulation. PdL-derived SP activated sensory neuron-like cells via the high affinity receptor NK1R. *Vice versa*, activated sensory neurons triggered a pro-inflammatory response of PdL cells in a SP/NK1R-dependent manner, thereby promoting osteoclast activation. These results significantly expand the current understanding of the interrelation between tissue inflammation, bone remodeling and pain during orthodontic treatment.

At earlier phases of orthodontic treatment, increasing SP levels in gingival cervical fluids have already been reported, with peak concentrations being reached 24 h after treatment was started (Shetty et al., 2020; Yamaguchi et al., 2009). More specifically, increased SP levels have been located in the PdL and dental pulp during simulated orthodontic tooth movement in rats and cats, with nociceptive sensory neurons suspected as origin (Nicolay et al., 1990; Norevall et al., 1995). Our study now demonstrates that also PdL fibroblasts can be considered as a source of SP in the context of orthodontic treatment. Our results show an increased expression of the SP precursor protein TAC1 and a secretion of SP by compressed PdLFs, peaking 24 h after loading. This is in line with studies on dental pulp fibroblasts (DPFs) of orthodontic patients revealing significantly enhanced SP expression after 24 h of applied orthodontic forces (Caviedes-Bucheli et al., 2021). Furthermore, Caviedes-Bucheli et al. (2021) reported a significantly higher level of SP expression in DPFs of occlusal trauma sides where additional orthodontic forces were applied. This further supports our finding of a force intensity-depending increase in TAC1 expression and SP secretion by PdLFs. Thus, besides its neuronal release, SP secreted by PdL fibroblasts may contribute to diverse modulatory processes during orthodontic tooth movement.

Neuron-released SP promotes neurogenic inflammation and contributes to tissue and bone remodeling by modulating the activity of diverse cell types (Navratilova and Porreca, 2019). Our results indicate that PdLF-derived SP may also activates sensory neurons via NK1R, as evidenced by classical neuron activity parameters such as increased morphological complexity, enhanced cFOS levels, and elevated calcium influx. Robust NK1R expression in differentiated SH-SY5Y sensory-like neurons as well as in sensory neurons of the trigeminal nerve have been previously demonstrated (Butler et al., 2018; Edvinsson et al., 2021). In this context, NK1R activation by SP could enhance the excitability of neurons in the spinal cord and brain regions involved in pain processing such as the amygdala and dorsal horn (Tang et al., 2007; Tillisch et al., 2012). Thereby, activation of sensory neurons by SP may sensitize these cells by reducing their activation threshold and contributing to pain hypersensitivity under pathological conditions (Moraes et al., 2014; Park et al., 2010). Additionally, SP could stimulate other sensory neuron populations, including those involved in itch sensation (Azimi et al., 2017). While NK1R is recognized as the primary receptor for SP, evidence suggests that under high SP concentrations, for example during intense mechanical stress, additional neurokinin receptors (NK2R and NK3R) may become activated (Chakraborty et al., 2011; Nguyen et al., 2023). We did not analyze the expression or function of additional receptors in our sensory neuron model. Since selective blocking of NK1R did not completely abolish the activation of sensory neurons by stressed hPdLFs, alternative receptors or other mediators may contribute to neuron activation, which could be analyzed in future studies. In this context, enhanced ATP release from stressed PdL cells activates nociceptive signaling pathways, contributing to pain perception during orthodontic treatment (Mizuhara et al., 2020). Furthermore, inflammatory cytokines secreted by compressed PdL fibroblasts, such as Il-1β, IL-6, TNF-α, and prostaglandin E2 (PGE2), may amplify pain signaling in part via stimulating microglia-neuron interactions (König et al., 2021; Leung and Cahill, 2010; St-Jacques and Ma, 2014). Moreover, chemokines such as CCL2 and CXCL8, which are secreted by PdL cells in response to mechanical stimulation (Madureira et al., 2012; Stemmler et al., 2021), have been implicated in modulating neuronal excitability (Belkouch et al., 2011; Cunha et al., 1991). Additionally, neurotrophic factors such as nerve growth factor (NGF) are implicated in modulating nociceptive transmission and promoting neurogenic inflammation (Meng et al., 2023; O’Hara et al., 2009). These findings underscore the multifaceted nature of neuropeptide signaling under mechanical stress.

While our study identifies a novel non-neuronal source of SP via stressed PdL fibroblasts, extensive research has already documented the pivotal role of sensory neuron-derived SP in tissue and bone remodeling as well as pain processing (Li et al., 2020; Navratilova and Porreca, 2019), also in the context of orthodontic treatment (An et al., 2019; Wang et al., 2024). Consistent with these findings, our data demonstrated that PdL fibroblasts exhibited a significant pro-inflammatory response with increasing expression levels of genes encoding IL-1β, IL-6, COX2 and TNF-α when exposed to SP-enriched conditioned medium derived from capsaicin-activated sensory-like SH-SY5Y neurons. Supporting our findings, previous research has specifically shown that SP stimulates the expression of pro-inflammatory cytokines not only in various immune cells (Mashaghi et al., 2016), but also in dental pulp fibroblasts (Yamaguchi et al., 2004; Yamaguchi et al., 2008). In this context, Yamaguchi et al. (2004, 2008) demonstrated that SP stimulation elicited a time- and concentration-dependent upregulation of IL-1β, IL-6, and TNF-α in human dental pulp fibroblasts obtained from both healthy individuals and patients with severe apical root resorption following orthodontic treatment. We hypothesize that SP, whether from neurons or PdL cells, may act as a local enhancer of inflammatory processes in the periodontal ligament during OTM. In addition, SP might also have autocrine effects, which may need to be clarified in future studies. Nevertheless, the enhanced inflammatory response of SP-stimulated PdL cells may also further sensitize adjacent nociceptive neurons and thus contribute to elevated pain perception during orthodontic tooth movement. Additional studies on the molecular mechanisms of SP-mediated inflammatory enhancement are required to fully elucidate the role of SP in the inflammatory signaling of PdL cells and nociceptive neurons during OTM.

Our findings indicate that SP promotes osteoclastogenesis by modulating the mechanoresponse of local PdL fibroblasts. In response to orthodontic forces, these cells are important regulators of osteoclast activity through the increased secretion of RANKL, which promotes osteoclast differentiation (Yamaguchi, 2009). When stimulated with SP-enriched conditioned medium of sensory-like neurons, PdLFs exhibited a significant up-regulation of *RANKL* and an increased *RANKL*/*OPG* ratio. Functionally, this shift in signaling was associated with an enhanced differentiation of osteoclasts. These findings corroborates the results of Yamaguchi et al. (2008) that demonstrated an increased number of TRAP-positive, multinucleated osteoclasts and resorption pits on dentin slice surface when precursors were stimulated with conditioned medium of SP-stimulated DPFs. This suggest that SP can shift the local microenvironment toward a pro-resorptive state by modulating the mechanoresponse of PdL cells. The enhanced RANKL level is likely to facilitate the recruitment and differentiation of osteoclast precursors, which is essential for bone resorption during orthodontic treatment. Furthermore, emerging evidence suggests that SP may also directly influence osteoclast precursors by activating intracellular signaling cascades, such as the NF-κB and MAPK pathways, promoting cell fusion and resorptive activity (Sohn, 2005; Wang et al., 2009). Collectively, our observations support the concept in which SP modulates inflammatory responses, as well as contributes to bone resorption by promoting osteoclastogenesis. In addition to the direct activation of immune cells and osteoclast precursors by SP, we now demonstrate an indirect pathway via the stimulation of PdL cells. The bidirectional release of SP from both sensory neurons and PdL fibroblasts has important biological implications. Such dual signaling may create a positive feedback loop in which nociceptive signaling and pain perception are enhanced while simultaneously stimulating the pro-inflammatory and pro-resorptive mechanoresponse of PdLFs. Therefore, this circuit may be important for fine-tuning local inflammatory and bone resorption activity during orthodontic tooth movement. In addition, the constant interplay between neuronal and fibroblastic SP release could lead to sensitization of the nociceptive pathways, potentially exacerbating pain during orthodontic treatment. Though, in animal studies, the highest SP levels were observed 24 h after force application, with a decline at later time points (Nicolay et al., 1990; Norevall et al., 1995). This indicates that SP is particularly important in the early phase of orthodontic tooth movement, which is characterized by pronounced mechanical compression of the PdL and the recruitment and activation of osteoclasts (Zainal Ariffin et al., 2011). Dysregulation of this bidirectional signaling with an excessive activation may lead to enhanced inflammatory response of the PdL and excessive bone resorption. As a result, hyperinflammatory/-resorptive responses may increase risks and support adverse effects during orthodontic treatment such as periodontal tissue loss, impaired tooth stability and chronic pain (Yamaguchi and Fukasawa, 2021).

While our *in vitro* model provided valuable insights into the role of PdLF-derived SP in modulating inflammatory responses and osteoclastogenesis, several limitations must be acknowledged. The simplified cell culture system does not fully replicate the complex *in vivo* environment of the periodontal ligament. Nevertheless, we applied 24 h of compressive force, which is the *in vivo* peak of pain sensation and correlated with maximal SP secretion observed in other studies (Nicolay et al., 1990; Norevall et al., 1995). Moreover, we only stimulated neuronal processes, reflecting the *in vivo* situation where the soma is located in the trigeminal ganglion. However, direct neuronal inputs might be essential for neuroimmune interactions between different cells, which could be addressed by co-cultures in future experiments. We cannot completely exclude residual NK1R activity in differentiated SH-SY5Y neurons due to potential cell type-specific differences when using aprepitant. However, due to its high affinity (Hale et al., 1998), we assume that the aprepitant concentration we used should have reliably blocked the activity of the NK1R. Finally, limitations by donor specificity might also impact the results. Although the SH-SY5Y cells were derived from a single patient, PdLFs were obtained from pooled donors of different gender and age to maximize variability. Nevertheless, donor-related factors such as age and gender may influence the characteristics and functional behavior of periodontal ligament (PdL) fibroblasts. For instance, different hormone levels as well as aging seem to influence PdL cell characteristics and functionality (Quast et al., 2021; Sawa et al., 2000). In addition, SP expression and activity are also influenced by both age and sex (Cetinkaya et al., 2020; Marco et al., 2018). Although we used a relatively heterogeneous pool of PdLFs from four donors different in origin, gender and age, we cannot fully rule out potential implications of donor variability.

In conclusion, our study revealed that periodontal ligament fibroblasts act as a novel non-neuronal source of substance P. Under a 24-h compressive stress corresponding to the peak in pain sensation, PdLFs secrete maximal levels of SP activating sensory neurons. Conversely, SP released from stimulated sensory neurons induces a pro-inflammatory and osteoclastogenic signaling by PdL fibroblasts. These results highlight the relevance of a dynamic intercellular interaction between PdL cells and sensory neurons that control the complex remodeling processes and pain sensitivity during orthodontic tooth movement.

## Data Availability

The raw data supporting the conclusions of this article will be made available by the authors, without undue reservation.

## Footnotes

1
https://imagej.net/software/fiji/downloads
